# The 2014 Minority Health and Health Disparities Grantees’ Conference

**DOI:** 10.3390/ijerph13010057

**Published:** 2015-12-22

**Authors:** Mark Edberg, Barbara E. Hayes, Valerie Montgomery-Rice, Paul B. Tchounwou

**Affiliations:** 1 Department of Anthropology, Columbian College of Arts & Science, George Washington University, 2110 G St. NW, Washington, DC 20052, USA; medberg@gwu.edu; 2 College of Pharmacy and Health Sciences, Texas Southern University, Gray Hall, 3100 Cleburne Street, Houston, TX 77004, USA; hayes_be@tsu.edu; 3 Morehouse School of Medicine, 720 Westview Drive SW, Atlanta, GA 30310, USA; vmrice@msm.edu; 4 RCMI Center for Environmental Health, College of Science, Engineering and Technology, Jackson State University, 1400 Lynch Street, Box 18750, Jackson, MS 39217, USA

## Abstract

Health disparities have been defined as a particular type of health difference closely linked with social, economic and/or environmental disadvantage. The National Institute on Minority Health and Health Disparities (NIMHD) at the National Institutes of Health, has a comprehensive portfolio of grants that fund scientific research to improve racial/ethnic minority health and eliminate health disparities. The *2014 Minority Health and Health Disparities Grantees’ Conference* highlighted excellence and innovation in biological, environmental, sociocultural, clinical and behavioral research supported by NIMHD. This special issue of the *International Journal of Environmental Research and Public Health* includes peer-reviewed publications from investigators who participated in this conference.

The 2014 Minority Health and Health Disparities Grantees’ Conference was held 1–3 December, in National Harbor, Maryland. The theme of the symposium, *“Transdisciplinary Collaborations: Evolving Dimensions of the United States and Global Health Equity”* called attention to the need for and power of collaborations across disciplines to improve minority health, reduce and eliminate health disparities and achieve health equity for all. The scientific program included general, and concurrent sessions, panel discussions and poster sessions. The conference also included specialized technical workshops; workshops on collaboration, scientific leadership, grantsmanship, and mentoring; and meet the expert sessions focused on career development for early stage investigators planned in collaboration with NIH staff. The conference offered unparalleled opportunities for networking and exchanging ideas, forging new scientific collaborations, resource sharing and opportunities for specialized training.

Health disparities have been defined as a particular type of health difference closely linked with social, economic, and/or environmental disadvantage. Hence, health disparities adversely affect groups of people who have systematically experienced greater obstacles to health based on their ethnicity or race, gender or sex, age, religion, socioeconomic status, mental health, physical disability, or other relevant characteristics [1]. Health disparities are not one phenomenon fixed in time, but evolve as circumstances and demographics change. With this as a backdrop, the conference keynote lectures and panels focused on four major themes:

- *State of the Science in Achieving Global Health Equity-Past and Present Advances and Future Directions*-highlighting best practices in biomedical, behavioral, population and health policy research that contribute to improvements in US and global health equity among populations who carry the burden of health disparities. Collaborative, sustainable, replicable and culturally appropriate research promoting prevention, reduction and elimination of heath disparities were also discussed;
- *Achieving Health Equity Through a Population Health Research Paradigm*-highlighting effective population health interventions that are often multi-sectorial and community engaged;
- *Transdisciplinary Social, Behavioral and Clinical Approaches for Understanding and Achieving Health Equity in Cardiovascular, Cerebrovascular, and Related Peripheral-Vascular Diseases*-highlighting ethnic- and racial-specific variations and determinants underlying health disparities in cardiovascular, cerebrovascular and related peripheral-vascular diseases; and
- *Transdisciplinary Collaborations: A Call to Action*-highlighting evidenced-based transdisciplinary research approaches to reduce health disparities, and the strategies to promote diversity in the biomedical research workforce.

In addition to keynote lectures and panels, 1364 abstracts were presented in thirty concurrent scientific sessions and three poster sessions. Research presented spanned a variety of disease areas-cardiovascular, diabetes, obesity, cancer, mental health, infectious disease, stroke and other diseases that disproportionately impact health disparity populations. Studies examined the etiology of disparities; the intersection of biological and behavioral risk factors, the physical environment, social determinants of health, and public health and health care system factors in addressing health disparities; and multi-level community interventions and other population-based studies to improve minority health and reduce health disparities. 

This special issue of the *International Journal of Environmental Research and Public Health* is dedicated to the publication of selected peer-reviewed manuscripts resulting from conference presentations. Highlights on a few of these research papers are as follows:

Carriere and collaborators [2] studied the role of glyceollin I in the treatment of Letrozole resistant breast cancer. They found that letrozole-resistance increased Zinc Finger E-Box Binding Homeobox 1 (ZEB1) expression by about 4 fold, while glyceollin I treatment caused a 4-fold reduction. Immunofluorescence analyses resulted in glyceollin I-induced increase and decrease in E-cadherin and ZEB1, respectively. They pointed out that the effects of glyceollin I were mediated in part by inhibition of ZEB1, thus indicating therapeutic potential of glyceollin I in targeting epithelial to mesenchymal transition in letrozole resistant breast cancer.

Salto and collaborators [3] conducted a study to characterize the effect of alanine to threonine amino acid substitution at codon 54 (*Ala54Thr*) polymorphism of the fatty acid binding protein 2 (FABP) on HDL cholesterol in Mexican-Americans with type 2 diabetes (T2D). They found that the Thr54 allele carriers who were heterozygous or homozygous for the threonine-encoding allele had lower HDL cholesterol and higher triglyceride levels at baseline compared to the Ala54 homozygotes. They concluded that the *Ala54Thr* polymorphism of FABP2 modulates HDL cholesterol in Mexican-Americans with T2D and that Thr54 allele carriers may be responsive in interventions that include dietary changes.

Shtraizent and collaborators [4] performed gain-of-function studies to investigate the role played by p53 gene mutation in breast cancer disparity among African American (AA) patients with triple negative breast cancer (TNBC). Using impedance-based real-time analysis they correlated the expression of mtp53 R248Q with increased cell deformability. They pointed out from their *in-vitro* study results that targeting the gain-of-function pathways may improve treatment efficacy when R248Q mtp53 proteins are expressed in TNBC.

Miranda-Diaz and collaborators [5] investigated the barriers for compliance to breast, colorectal and cervical cancer screening tests among Hispanic patients, and reported that the major reasons for avoiding mammography and PAP tests as having a busy schedule, fear, and feeling uncomfortable during the procedure. 

In a retrospective epidemiologic study, Holmes and collaborators [6] investigated the prevalence of childhood cancer in the State of Delaware and reported a cumulative incidence of 234 per 100,000 children. Most importantly, these rates varied by race—blacks (273 per 100,000) and whites (189 per 100,000), and by gender—boys (237 per 100,000) and girls (230 per 100,000). Disparities in overall childhood cancer distribution underscored the need for cancer-specific health education, awareness and prevention programs in reducing the observed disparities in Delaware. In a separate cross sectional study design Holmes and collaborators [7] also assessed the association as well as the racial/ethnic heterogeneity between total cholesterol (TC) and pediatric overweight/obesity. They reported that a significant racial variability in TC was observed, and its levels correlating well with children’s BMIs.

Using the Jackson Heart Study as a model, Addison and collaborators [8] highlighted the importance of building a collaborative health promotion partnership that effectively employs principles of community-based participatory research (CBPR) to address population health. They recommended that academic institutions must reach out to local community groups and together address local health issues that affect the community. Townsend and collaborators [9] conducted a CBPR to better understand and address the disproportionate burden of type-2 diabetes and related complications among Native Hawaiians compared to all other groups in Hawaii (e.g., Whites, Japanese, Korean). Their study findings stressed the importance of securing the thrust of communities for research participation, and engagement in health assessments and disease control and prevention.

Using both GIS and regression analysis models, Mathis and collaborators [10] conducted a study to examine the association between neighborhood environment and self-rated health (SRH) among urban older adults. They reported that seniors who had poor SRH were 21% more likely to report fear of crime than seniors with excellent SRH. They recommended that environmental influences in neighborhoods with poor SRH be controlled in order to improve health and reduce disability.

We would like to thank the conference organizing committee, scientific review committee, other conference committees and staff, and the National Institute on Minority Health and Health Disparities leadership and staff for their contributions to making the conference a success and publication of this special issue possible.

## References

[1] United States Department of Health and Human Services The Secretary’s Advisory Committee on National Health Promotion and Disease Prevention Objectives for 2020. Phase I Report: Recommendations for the framework and format of Healthy People 2020. http://www.healthypeople.gov/sites/default/files/PhaseI_0.pdf.

[2] Carriere P., Llopis S.D., Naiki A.C., Nguyen G., Phan T., Nguyen M.M., Preyan L.C., Yearby L., Pratt J., Burks H. (2016). Glyceollin I reverses epithelial to mesenchymal transition in letrozole resistant breast cancer through ZEB1. Int. J. Environ. Res. Public Health.

[3] Salto L.M., Bu L., Beeson W.L., Firek A., Cordero-MacIntyre Z., De León M. (2016). The Ala54Thr polymorphism of the fatty acid binding protein 2 gene modulates glycemic control improvements in Mexican-Americans with Type 2 diabetes. Int. J. Environ. Res. Public Health.

[4] Shtraizent N., Matsui H., Polotskaia A., Bargonetti J. (2016). Hot spot mutation in TP53 (R248Q) causes oncogenic gain-of-function phenotypes in a breast cancer cell line derived from an African American patient. Int. J. Environ. Res. Public Health.

[5] Miranda-Diaz C., Betancourt E., Ruiz-Candelaria Y., Hunter-Mellado R.F. (2016). Barriers for compliance to breast, colorectal, and cervical screening cancer tests among Hispanics patients. Int. J. Environ. Res. Public Health.

[6] Holmes L., Vandenberg J., McClarin L., Kirk Dabney K. (2016). Epidemiologic, racial and healthographic mapping of Delaware pediatric cancer: 2004–2014. Int. J. Environ. Res. Public Health.

[7] Holmes L., LaHurd A., Wasson E., McClarin L., Dabney K. (2016). Racial and ethnic heterogeneity in the association between serum lipid and pediatric obesity. Int. J. Environ. Res. Public Health.

[8] Clifton Addison C., Brenda Campbell Jenkins B.C., Odom D., Fortenberry M., Wilson G., Young L., Antoine-LaVigne D. (2016). Building collaborative health promotion partnerships: The Jackson Heart Study. Int. J. Environ. Res. Public Health.

[9] Townsend C.K.M., Dillard A., Hosoda K.K., Maskarinec G., Maunakea A., Yoshimura S.R., Hughes C., Palakiko D-M., Kekauoha B.P., Kaholokula J.K. (2016). Community-based participatory research integrates behavioral and biological research to achieve health equity for Native Hawaiians. Int. J. Environ. Res. Public Health.

[10] Arlesia Mathis A., Rooks R., Kruger D. (2016). Improving the neighborhood environment for urban older adults: Social context and self-rated health. Int. J. Environ. Res. Public Health.

